# Rural residents’ willingness to utilize primary care and its influencing factors in the context of graded diagnosis and treatment: a study in Deqing, China

**DOI:** 10.3389/fpubh.2024.1478686

**Published:** 2024-12-02

**Authors:** Meng Gao, Xueqing Zhu

**Affiliations:** School of Economics and Management, Zhejiang Ocean University, Zhoushan, Zhejiang, China

**Keywords:** graded diagnosis and treatment, primary care, theory of planned behavior, structural equation modeling, China

## Abstract

**Background:**

Graded diagnosis and treatment is a key component in advancing healthcare system reforms and establishing a foundational healthcare framework. Primary care serves as the cornerstone of this system. Understanding the willingness to seek primary care and the factors influencing it can enhance primary care utilization and address the challenges of “difficult access to healthcare” and “high medical costs.”

**Methods:**

This study, based on data from 415 rural residents in Deqing County, Zhejiang Province, analyzes their willingness to seek primary care and the factors influencing it using the Theory of Planned Behavior and structural equation modeling.

**Results:**

The results of the study show the following: ① Attitude toward behavioral, subjective norm, and perceived behavior control significantly affect willingness to seek primary care, with attitude toward behavior having the greatest influence. ② Regarding attitude toward behavior, residents’ evaluation of treatment costs in primary care, the medical environment, and trust in doctors significantly impact their willingness to seek primary care. ③ In terms of subjective norms, the level of support from others, as well as the recognition of graded diagnosis and treatment systems and family doctor contracting services, positively influence the willingness to use primary care. ④ For perceived behavioral control, residents’ perception of personal and environmental factors affects their willingness to make a first visit to primary care facilities.

**Conclusion:**

The study recommends strengthening the awareness of the necessity of primary care among rural residents, improving the experience at the primary level, stimulating the desire for primary care, and increasing the frequency of primary care utilization.

## 1 Introduction

In 2016, “Healthy China” was established as a national strategy, with a core focus on prioritizing people’s health and emphasizing grassroots healthcare. The development of healthy villages is an important component of achieving the “Healthy China” goals and plays a significant role in rural revitalization. Graded diagnosis and treatment serve as a key approach to strengthening healthcare at the grassroots level (1).

Population aging and rapid urbanization have transformed people’s health perceptions, leading to an “inverted pyramid” demand for medical services, where residents increasingly seek high-level medical care. Although healthcare reforms have increased investments in primary care institutions and system infrastructure, the goal of strengthening the grassroots level has not yet been fully realized (2).

In 2015, the General Office of the State Council issued the “Guiding Opinions on Promoting the Construction of Graded Diagnostic and Treatment Systems,” which, for the first time, explicitly emphasized enhancing the capacity of primary healthcare institutions and guiding residents toward seeking treatment through a graded system (3). In 2023, the General Office of the Central Committee of the Communist Party of China and the General Office of the State Council issued further opinions on improving the medical and healthcare systems. These opinions proposed strengthening the division of labor and cooperation, promoting graded diagnosis and treatment, and building a comprehensive, high-quality, and efficient healthcare system with Chinese characteristics.

The foundation and prerequisite for effectively advancing a graded diagnosis and treatment system depend on whether primary care can successfully fulfill its role as “health gatekeepers” (4).

Based on data from the *China Health and Health Statistics Yearbook* (2022), the number of visits to primary care organizations decreased instead of increasing from 2017 to 2021, with the proportion of visits decreasing from 54.12% in 2017 to 50.17% in 2021. By the end of 2021, the share of inpatient services in primary care organizations decreased from 18.21% in 2017 to 14.52% (5). A study by Xu found that 63.8% of residents in a community in Huai’an City were willing to seek primary care (6). Similarly, Anran et al. found that the willingness to use primary care services among residents of Dongguan City, Huizhou City, and Zhanjiang City was 59.02, 52.16, and 48.52%, respectively (7). In contrast, Han et al., analyzing data from 64,028 respondents in the 2017 National Migrant Population Dynamics Monitoring, found that only 17.79% were willing to choose primary care (8).

According to Yu et al., the scarcity of primary healthcare resources and the diminishing healthcare capacity are major constraints on the development of primary healthcare institutions (9). Wu et al. believe that the government should take a leading role in optimizing the diagnosis and treatment model, improving the quality of medical services, and better meeting citizens’ health needs (10).

In this context, conducting an in-depth study on rural residents’ willingness to seek primary care and the factors influencing their decisions is crucial. Such research will further promote the implementation of the graded diagnosis and treatment system, contributing to the goal of a “healthy China.”

Research by domestic and international scholars on the factors influencing willingness to seek primary care mainly focuses on two aspects:

On the one hand, internal factors are believed to have a significant impact on individuals’ willingness to attend primary. These factors mainly include gender (11), age (12), health status (13), average annual disposable income (14), educational attainment (15), level of knowledge of the graded diagnosis and treatment system (16), the level of understanding of the family doctor system (17), the level of trust in the service capacity of primary care institutions and other influencing factors (18).

On the other hand, external factors also affect residents’ willingness to choose primary care. These factors mainly include the proximity of medical institutions (19), health insurance reimbursement rates (20), treatment fees at primary care institutions (21), the medical environment (22), family doctor contracting (23), primary care infrastructure (24), and waiting time and other influencing factors (25).

In addition, research by Pavel Alexandru, Machado Sergio, and other scholars has found that subjective cognitive abilities can impact perceptions of illness and medical treatment (26, 27). Zhao et al. found that in the interpersonal networks of rural communities, residents who receive support from family and friends are more willing to seek primary care (28).

Overall, existing studies have explored the factors influencing primary care utilization in some regions, with some studies analyzing and validating key factors using logistic regression models. These efforts have laid the groundwork for this paper. However, there remains a need to expand and deepen the current research.

First, rural residents have unique characteristics, and the factors influencing their healthcare decisions are not necessarily the same as those in urban areas. Ai et al. conducted a questionnaire survey involving 675 rural residents and found that these rural residents tend to perceive the severity of diseases as too high while having a low perception of the capabilities of primary care institutions (29). Strengthening the analysis of the influencing factors specific to rural residents can help accelerate the deeper implementation of graded diagnosis and treatment.

Second, while some studies have analyzed the relationship between influencing factors using logistic regression models—such as the study by Chen Lijiang and other scholars, which concluded that age, household registration type, and support for two-way referrals are significant factors affecting patients’ first consultation choice (30)—logistic regression has its limitations: It can only address linear relationships and cannot capture the complex patterns within the data. Therefore, a new analytical model is needed to reveal both direct and indirect influences among factors and to explore the evolving characteristics of rural residents’ willingness to seek primary care, considering multiple interlinked factors.

Increasing the rate of primary care utilization among rural residents and promoting the system of graded diagnosis and treatment remains a significant challenge. How do various factors affect the formation of rural residents’ willingness to seek primary care? What factors are the most significant? What mechanisms underlie the development of this willingness?

Deqing County, as a pilot area for the construction of the county medical community, provides a typical case study, making it an ideal research object. This study constructs a comprehensive theoretical hypothesis model based on the Theory of Planned Behavior, taking into account both internal and external factors. Structural equation modeling is employed to analyze the detailed connections between rural residents’ willingness to seek primary care and the influencing factors, with the aim of enriching the theoretical foundation and informing relevant policy formulation. The primary healthcare institutions examined in this study mainly refer to township health centers and village clinics.

## 2 Materials and methods

Our study approval received approval from the Ethics Committee of the School of Economics and Management at Zhejiang Ocean University. In accordance with the ethical principles outlined in the Declaration of Helsinki, all participants provided informed consent before participating in the study. The anonymity and confidentiality of the participants were assured, and participation was entirely voluntary.

### 2.1 Theoretical foundations and research hypotheses

#### 2.1.1 Theoretical foundation

The Theory of Planned Behavior (TPB) originates from the Theory of Reasoned Action (TPA), proposed by American scholars Paul Fishbein and Icek Ajzen in 1975, which demonstrated strong explanatory power in studying individual intentions and behaviors through the constructs of attitude toward the behavior (AB) and subjective norm (SN) (31). In 1985, Ajzen expanded the Theory of Reasoned Action by introducing perceived behavioral control (PBC), positing that while human behavior is primarily determined by behavioral intentions, these intentions are influenced by AB, SNs, and PBC (32).

Attitude toward the behavior reflects an individual’s preference for a particular behavior; subjective norms represent the level of pressure an individual perceives from individuals or organizations around them to either engage in or avoid a behavior (33); and perceived behavioral control refers to the perceived level of resources or capabilities an individual believes they have to perform a specific action (34).

In 2003, Icek Ajzen further refined these three constructs into five influencing factors: evaluation of results (ER), result belief (RB), normative belief (NB), motivation to comply (MC), and control belief (CB) (35). This refinement not only enhanced the explanatory power of the model for understanding influences on individual behavioral intentions but also improved its effectiveness in predicting such intentions (36).

#### 2.1.2 Research hypotheses

According to the Theory of Planned Behavior, this study hypothesizes that residents’ willingness to seek primary care within the context of a graded diagnosis and treatment system is influenced by three main factors: attitude toward the behavior, subjective norm, and perceived behavioral control. Additionally, these three factors are further influenced by five components: evaluation of results, result belief, normative belief, motivation to comply, and control belief.

**Figure 1:**
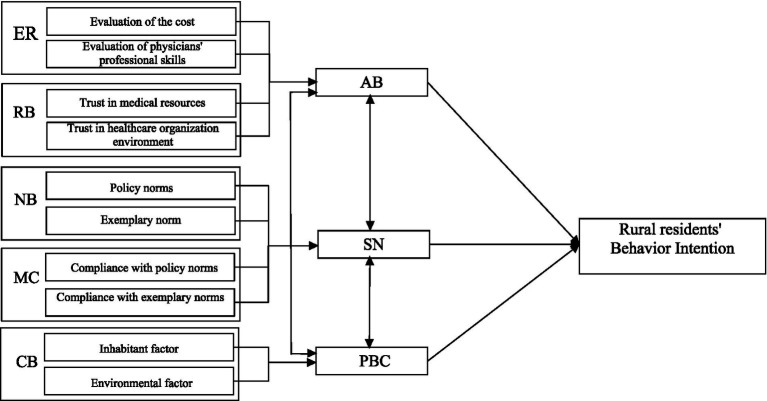
A hypothesized model of factors affecting rural residents’ behavioral intentions.

Based on this framework, a hypothesized model of the factors affecting rural residents’ willingness to seek primary care is proposed (Figure 1), leading to the following research hypotheses:

H1: Attitude toward the behavior has a positive impact on rural residents’ behavioral intentions.

H2: Evaluation of results has a positive impact on rural residents’ attitudes toward the behavior.

H3: Result belief has a positive impact on rural residents’ attitude toward the behavior.

H4: Subjective norm has a positive impact on rural residents’ willingness to seek primary care.

H5: Normative belief has a positive impact on the subjective norm of residents.

H6: Motivation to comply has a positive impact on the subjective norm of residents.

H7: Perceived behavioral control has a positive impact on residents’ willingness to seek primary care.

H8: Inhabitant factors have a positive impact on the perceived behavioral control of residents.

H9: Environmental factors have a positive impact on the perceived behavioral control of residents.

### 2.2 Sample selection and data collection

The data for this study were obtained from a questionnaire survey assessing the willingness of rural residents in Deqing County, Zhejiang Province, to seek primary care. Two to three administrative villages were randomly selected from each of the eight towns and five streets, totaling 30 villages. Subsequently, 16 to 17 villagers were randomly chosen from each administrative village for participation in the study.

To verify the validity of the questionnaire, a pilot survey was conducted with 50 questionnaires. Based on the pilot results, six questions were removed due to respondent objections.

After revising the original questionnaire, a total of 482 questionnaires were distributed, with 447 collected. After excluding items from the pilot survey and responses with predominantly extreme answers, a final effective response rate of 86% was achieved, resulting in 415 valid questionnaires. The design of the questionnaire adhered to principles of scientific rigor, purposefulness, completeness, logical consistency, neutrality, rationality, and exclusivity. Table 1 shows the basic characteristics of the sample of rural residents.

**Table 1:** Basic characteristics of rural residents in the sample.

Character	Categorization	Number of people	Proportion (%)
Gender	Men	185	44.6%
	Women	230	55.4%
Age	Under 25	17	4.1%
	26–35 years old	29	7.0%
	36–45 years old	131	31.6%
	46–55 years old	152	36.6%
	Over 56 years old	86	20.7%
Educational background	Primary and below	122	29.4%
	Junior secondary schools	192	46.3%
	Senior secondary schools	57	13.7%
	undergraduate	34	8.2%
	Graduate students and above	10	2.4%
Average annual disposable income	More than 70,000 RMB	14	3.4%
	50,001–70,000 RMB	55	13.3%
	30,001–50,000 RMB	115	27.7%
	10,001–30,000 RMB	156	37.6%
	10000RMB and below	75	18.1%
Health status	Very unhealthy	22	5.3%
	Less healthy	31	7.5%
	general	194	46.7%
	Quite healthy	138	33.3%
	Very healthy	30	7.2%
Participation in basic medical insurance for urban and rural residents	Yes	370	89.2%
	No	45	10.8%

### 2.3 Questionnaire design

#### 2.3.1 Question item design and measurement method

In this study, the variable question items were developed based on the existing mature scales and research data on rural residents’ willingness to seek primary care at the grassroots level, ensuring the scientific validity of the questionnaire. The questionnaire consists of 10 latent variables and 39 observed variables, including behavioral intention, attitude toward the behavior, subjective norm, perceived behavioral control, evaluation of results, result belief, normative belief, motivation to comply, inhabitant factors, and environmental factors.

A five-level scale Likert scale measure was used for scoring (Strongly disagree = 1; Disagree = 2; Not sure = 3; Agree = 4; and Strongly agree = 5) (37). To minimize the impact of extreme values on the final measurement results, continuous numerical variables were transformed into categorical variables according to the specific research context, as shown in Table 2.

**Table 2:** Questionnaire measurement items and definitions.

Latent variable	Serial number	Observed variable
BI	Bi1	Intensity of residents’ willingness to choose primary care
	Bi2	Residents believe that primary care is in line with expectations
	Bi3	The strength of residents’ willingness to allow friends and relatives to choose primary care
AB	Ab1	Whether primary care organizations are beneficial for treatment
	Ab2	Whether primary care organizations can meet basic health care needs
	Ab3	Residents’ perceptions of the cost of primary care
ER	Ab4	Satisfaction with the reasonableness of the cost of treatment
	Ab5	Satisfaction with the level of medical care
	Ab6	Satisfaction with physician-patient communication
	Ab7	Satisfaction with physicians’ defense of patients’ rights and interests
RB	Ab8	Trust in doctors’ medical skills
	Ab9	Trust in the medical equipment
	Ab10	Trust in medicines
	Ab11	Trust in the medical environment
SN	Sn1	Extent to which access to places of care is entirely at the discretion of the residents
	Sn2	Others’ approval rating of residents’ choice of primary care for medical care
	Sn3	The extent to which residents are willing to listen to others in choosing primary care
NB	Nb1	Family support for primary care for residents
	Nb2	Friends’ support for primary care for residents
	Nb3	Residents’ level of recognition of the graded diagnosis and treatment system for primary care
	Nb4	Residents’ level of recognition of family doctor service contracts for primary care
MC	Mc1	Residents’ level of compliance with family support and non-support for primary care
	Mc2	Residents’ level of compliance with friend support and non-support for primary care
	Mc3	Residents’ level of compliance with the graded diagnosis and treatment system for seeking primary care
	Mc4	Residents’ level of obedience in family doctor service contracts asking for primary care
PBC	Pbc1	Primary care provides the convenience of seeking medical treatment
	Pbc2	Primary care medical insurance payment rate
	Pbc3	Level of knowledge of the residents about the graded diagnosis and treatment system
	Pbc4	Level of knowledge of the residents about the family doctor service
	Pbc5	Percentage of contracted family doctors
IPBC	Ipbc1	Age
	Ipbc2	Educational background
	Ipbc3	Average annual disposable income
	Ipbc4	Health status
EPBC	Epbc1	Distance to primary care
	Epbc2	Accessibility to primary care
	Epbc3	Length of time spent in primary care
	Epbc4	Environmental sanitation in primary care
	Epbc5	Infrastructure of primary health care institutions

#### 2.3.2 Description of questions

① Willingness to primary care. This study is based on Icek Ajzen’s research (38) and is tailored to the characteristics of rural residents’ willingness to seek initial consultation at the grassroots level. It includes three question items (Bi1-Bi3).

② Attitude toward the behavior: Drawing on the findings of Zhou et al. (39), Shen et al. (40), and other scholars, three questions were developed to assess attitude toward behavior (Ab1-Ab3), four questions for evaluation of results (Ab4-Ab7), and four questions for result belief (Ab8-Ab11).

③ Subjective norm. Based on the studies of Wang et al. (41), Liu et al. (42), and other scholars, a total of three items (Sn1-Sn3) were designed for a subjective norm, four items (Nb1-Nb4) for normative belief, and four items (Mc1-Mc4) for motivation to comply.

④ Perceived behavioral control: According to the research results of Tang et al. (43), Song et al. (44), and other scholars, a total of five questions were formulated for perceived behavioral control (Pbc1-Pbc5), four questions for inhabitant factors (Ipbc1-Ipbc4), and five questions for environmental factors (Epbc1-Epbc5).

### 2.4 Measurement and analytical methods

To study rural residents’ willingness to seek primary care and its influencing factors, this study constructs a hypothesized model based on the Theory of Planned Behavior (TPB) and utilizes Structural Equation Modeling (SEM). SEM, an extension of the general linear model, is a well-established data analysis method and has become a key tool for multivariate analysis (45).

Given the interconnection between rural residents’ willingness to seek primary care and influencing factors—such as latent variables like attitude toward the behavior, subjective norm, and perceived behavioral control—this research involves conducting a factor analysis and path analysis on the model of the influencing factors. The theoretical hypothesis model is converted into a statistical model, with fitting evaluation indicators and parameter estimation results used to assess the alignment between the hypothesized model and the data. Additionally, observed variables are employed to further explain multiple latent variables that cannot be directly measured. For these reasons, structural equation modeling (SEM) was selected as the research method (46). The use of SEM in analyzing rural residents’ willingness to seek primary care offers several advantages: Flexibility in measurement: The measurement model allows for greater flexibility in estimating the factor structure and relationships. Handling measurement error: It accounts for measurement errors in both latent and observed variables when analyzing multiple variables (47). Comprehensive path analysis: It assesses not only the strength of individual paths but also the overall fit of the model, thus providing a more accurate representation of the relationships indicated by the data (48).

Structural equation modeling consists of structural equations that explain the relationships among latent variables, as well as measurement equations that clarify the relationships between latent variables and their corresponding indicators. These equations are represented through three matrix equations as follows:

Structural equation:


(1) η=Βη+Γξ+ζ

Measurement equation:


(2) χ=Λxξ+δ

(3) $$y=\Lambda_{y}\eta +\varepsilon$$

In the structural Equation 1, Β η represents the influence relationship between the endogenous latent variables η, while Γ ξ describes the influence of the exogenous latent variable ξ on the endogenous latent variable (η). The term *ζ* denotes the residual term.

The measurement Equation 2 pertains to the exogenous latent variable, where x represents the exogenous observed variable, and *δ* is the measurement error vector for the exogenous observed variable.

Similarly, Equation 3 is the measurement equation for the endogenous latent variable, where y denotes the endogenous observed variable, and *ε* represents the measurement error vector for the endogenous observed variable (49).

In this study, the endogenous latent variables (*η*) include rural residents’ behavioral intentions, attitude toward the behavior, subjective norm, and perceived behavioral control. The exogenous latent variables (*ξ*) consist of an evaluation of results, result belief, normative belief, motivation to comply, inhabitant factor, and environmental factor.

## 3 Results

### 3.1 Data reliability and validity tests

#### 3.1.1 Reliability test

The questionnaire data were imported into SPSS 27.0 software, where the 39 observed variables were analyzed using factor rotation with the Varimax rotation. After excluding seven variables with factor loadings below 0.5 and factor question items numbering fewer than 2, the remaining 32 variables were retained for further analysis. The results indicated that the *KMO* value was greater than 0.7, and *Bartlett’s Test of Sphericity* was significant (*p* = 0.000), confirming that the scale was suitable for factor analysis.

The reliability of the questionnaire was subsequently tested, showing that the scales were reliable, with *Cronbach’s alpha* values greater than 0.6 (Table 3).

**Table 3:** Reliability test.

Latent variable	Cronbach’s alpha	Number of measurable variables
BI	0.849	3
AB	0.864	3
ER	0.897	3
RB	0.878	3
SN	0.855	3
NB	0.907	4
MC	0.902	4
PBC	0.871	3
resident factor	0.756	3
environmental factor	0.850	3

#### 3.1.2 Validity test

This study used a questionnaire scale constructed based on relevant domestic and international literature, a solid theoretical foundation, and the findings of the pilot investigation. As a result, the scale demonstrates good content and criterion validity. As indicated in Table 4, the factor loading coefficients for each variable are greater than 0.5 and highly significant (*p* = 0.000), indicating high data validity (50).

**Table 4:** Validity test.

Latent variable	Observed variable	Factor loading	S.E.	Mean	*P*-value
BI	Bi1	0.793	0.051	3.660	0.000
	Bi2	0.847	0.048	3.680	0.000
	Bi3	0.802	0.049	3.760	0.000
AB	Ab1	0.830	0.051	3.590	0.000
	Ab2	0.806	0.052	3.520	0.000
	Ab3	0.846	0.055	3.520	0.000
ER	Ab4	0.845	0.059	3.130	0.000
	Ab5	0.836	0.060	3.090	0.000
	Ab6	0.829	0.059	3.070	0.000
RB	Ab8	0.836	0.046	3.670	0.000
	Ab10	0.805	0.048	3.660	0.000
	Ab11	0.797	0.049	3.710	0.000
SN	Sn1	0.832	0.053	3.480	0.000
	Sn2	0.771	0.059	3.490	0.000
	Sn3	0.740	0.055	3.630	0.000
NB	Nb1	0.822	0.056	3.450	0.000
	Nb2	0.851	0.056	3.310	0.000
	Nb3	0.839	0.056	3.420	0.000
	Nb4	0.819	0.056	3.310	0.000
MC	Mc1	0.878	0.056	2.810	0.000
	Mc2	0.855	0.054	2.840	0.000
	Mc3	0.838	0.054	2.880	0.000
	Mc4	0.850	0.055	2.860	0.000
PBC	Pbc1	0.811	0.050	3.430	0.000
	Pbc2	0.854	0.053	3.480	0.000
	Pbc5	0.878	0.052	3.480	0.000
IBPC	Ipbc1	0.811	0.050	3.630	0.000
	Ipbc3	0.739	0.051	3.540	0.000
	Ipbc4	0.798	0.045	3.300	0.000
EPBC	Epbc1	0.803	0.056	3.530	0.000
	Epbc2	0.820	0.058	3.450	0.000
	Epbc3	0.836	0.056	3.410	0.000

The results demonstrate that the latent variables—rural residents’ behavioral intentions, attitude toward the behavior, subjective norm, perceived behavioral control, evaluation of results, result belief, normative belief, motivation to comply, inhabitant factor, and environmental factor—were effectively represented by their corresponding observed variables.

### 3.2 Model fitting results

**Figure 2:**
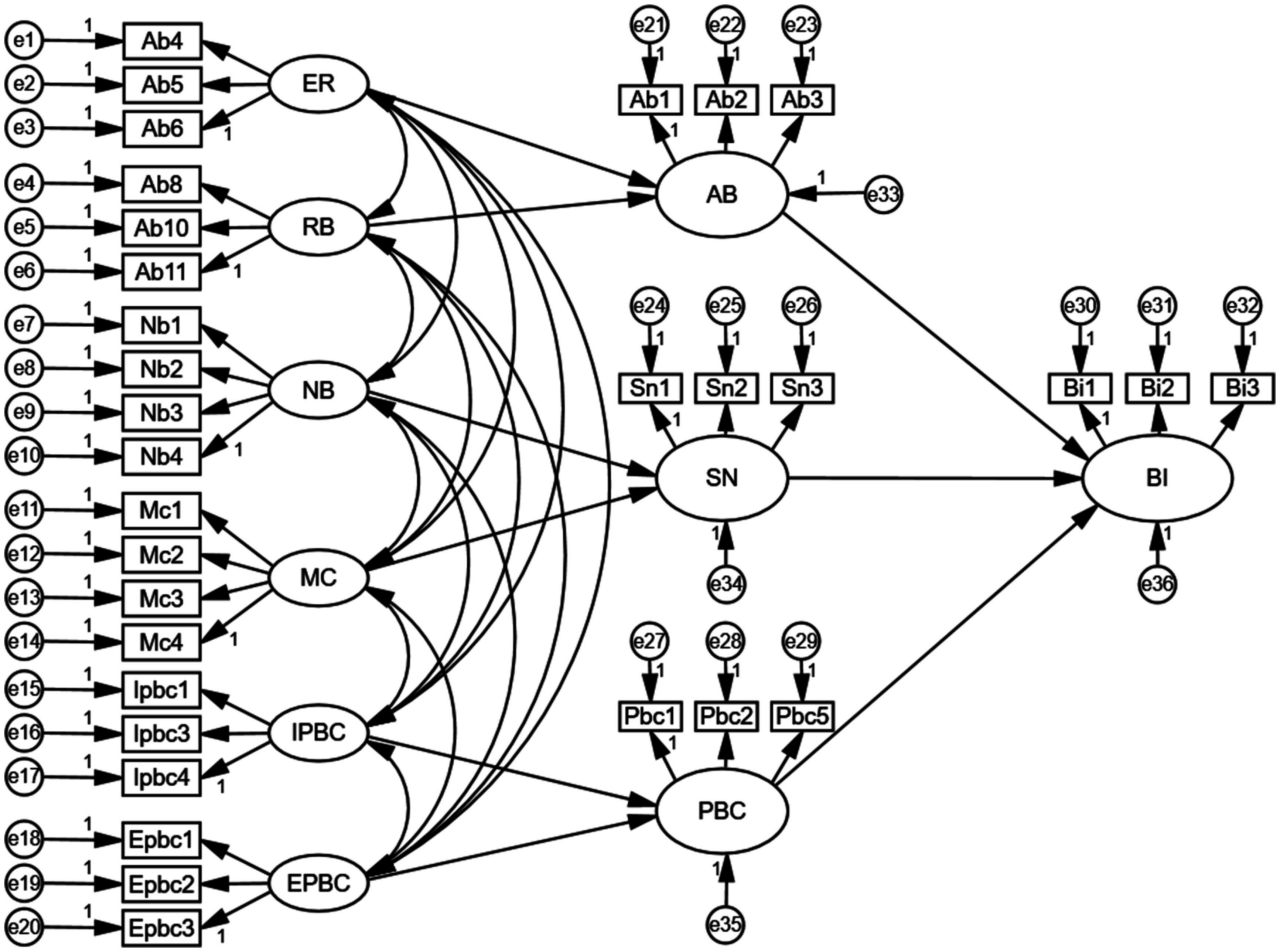
An initial path diagram of rural residents’ willingness to seek primary care.

Based on the results of the above-mentioned analysis, the SEM constructed in this study was tested for fitness using Amos22.0 software, and the initial model path is shown in Figure 2. The results indicated that the observable values for motivation to comply were low, and the path coefficients from motivation to comply to the subjective norm were not significant, demonstrating that hypothesis H6 is not valid. Consequently, the path from motivation to comply with subjective norms was removed. The model was then reanalyzed to obtain the relevant fit indices, results, and evaluation criteria (Table 5).

**Table 5:** Overall fitness test for structural equation modeling.

Index name		Evaluation criteria	Initial fit value	Result
Absolute fit index	x^2^/df	<3.0	2.084	Accept
	GFI	>0.8	0.894	Accept
	RMSEA	<0.08	0.051	Accept
	ECVI	<Saturated and independent model values	2.029	Accept
Relative fit index	NFI	>0.9	0.906	Accept
	IFI	>0.9	0.949	Accept
	TLI	>0.9	0.942	Accept
	CFI	>0.9	0.949	Accept
Information index	AIC	The smaller, the better	839.937	Accept
	PNFI	>0.5	0.796	Accept
	PCFI	>0.5	0.833	Accept

GFI denotes goodness-of-fit index; RMSEA denotes root mean square of approximation error; NFI denotes canonical fit index; IFI denotes incremental fit index; TLI denotes Tucker-Lewis index; CFI denotes comparative fit index; AIC denotes Akaike Information Criterion.

As shown in Table 5, all fitting indices meet the model’s acceptable criteria, which indicates that the theoretical model of the factors impacting rural residents’ willingness to seek primary care is a good fit.

### 3.3 Model corrections and determinations

**Figure 3:**
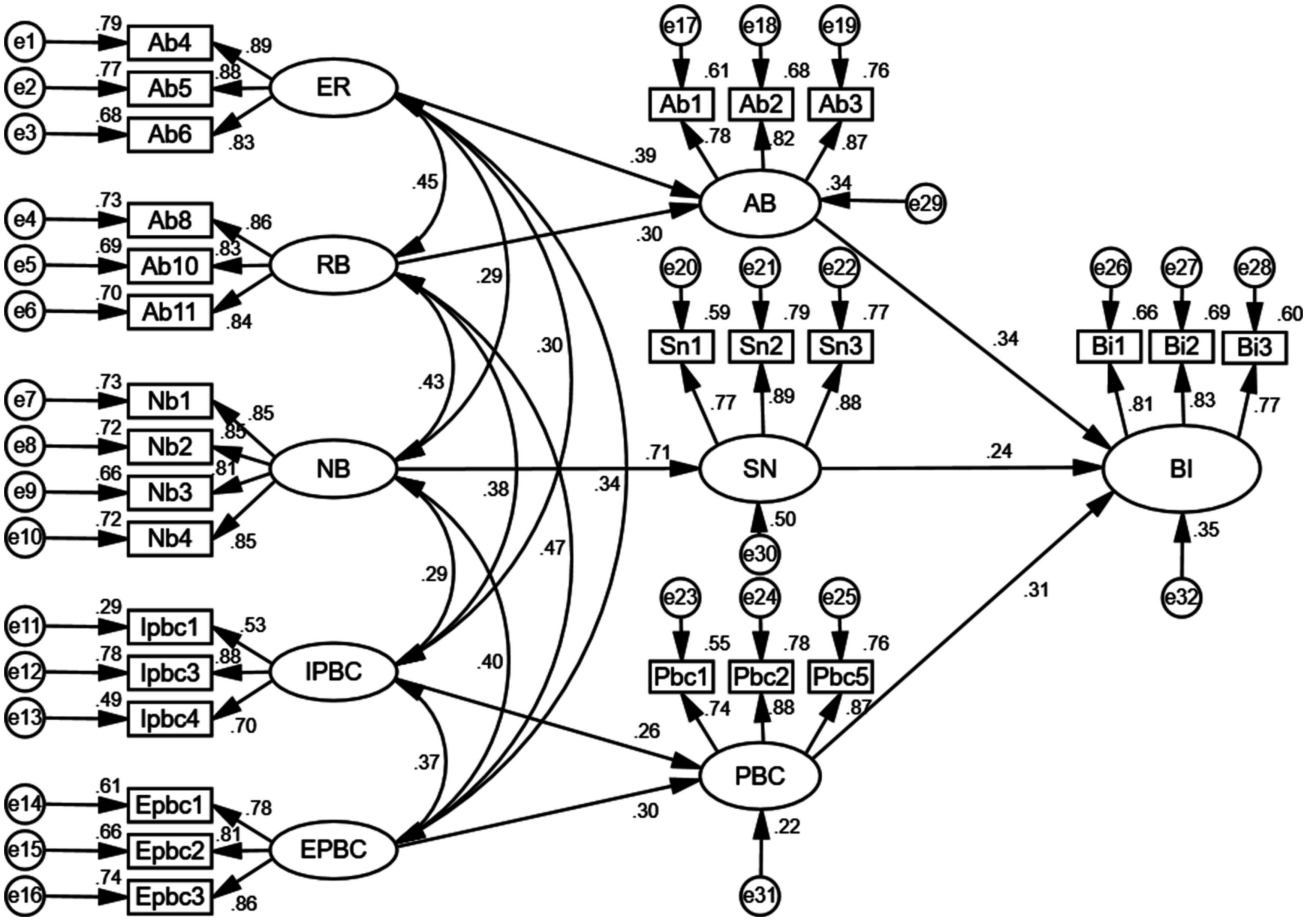
A final path map of rural residents’ willingness to seek primary care.

Considering that the path coefficient from motivation to comply with the subjective norm in the initial model of rural residents’ willingness to seek primary care was not significant, this path was removed to improve the overall model fit. The final paths obtained are shown in Figure 3. As shown in Figure 3, the theoretical hypotheses proposed in this study have been confirmed, resulting in an improved model fit.

### 3.4 Analysis of results

The results of the latent variable effects in the model, derived after processing the standardized sample data, are presented in Table 6. Table 7 shows the estimated coefficients for each path in the model. According to the *p*-values, the modified model path relationships are all significant at the 1% level. As shown in Table 6, the path coefficients of attitude toward the behavior, subjective norm, and perceived behavioral control on behavioral intention were 0.350, 0.244, and 0.342, respectively, indicating significant direct effects and confirming the validity of Hypotheses H1, H4, and H7.

**Table 6:** Standardized results of total, direct, and indirect effects between latent variables in the model.

Variables	Effect	EPBC	IPBC	NB	RB	ER	PBC	SN	AB	BI
PBC	Total effect	0.230	0.310	0	0	0	0	0	0	0
	Direct effect	0.230	0.310	0	0	0	0	0	0	0
	Indirect effect	0	0	0	0	0	0	0	0	0
SN	Total effect	0	0	0.610	0	0	0	0	0	0
	Direct effect	0	0	0.610	0	0	0	0	0	0
	Indirect effect	0	0	0	0	0	0	0	0	0
AB	Total effect	0	0	0	0.295	0.318	0	0	0	0
	Direct effect	0	0	0	0.295	0.318	0	0	0	0
	Indirect effect	0	0	0	0	0	0	0	0	0
BI	Total effect	0.079	0.106	0.149	0.103	0.112	0.342	0.244	0.350	0
	Direct effect	0	0	0	0	0	0.342	0.244	0.350	0
	Indirect effect	0.079	0.106	0.149	0.103	0.112	0	0	0	0

**Table 7:** Estimated coefficients for each path of the model.

Pathway relationship	Estimate	S.E.	C.R.	P
Bi1 ← BI	0.814			
Bi2 ← BI	0.829	0.057	16.838	***
Bi3 ← BI	0.772	0.057	15.933	***
AB→BI	0.340	0.055	6.389	***
Ab1 ← AB	0.781			
Ab2 ← AB	0.824	0.063	17.114	***
Ab3 ← AB	0.869	0.067	17.781	***
ER → AB	0.386	0.048	6.687	***
Ab4 ← ER	0.886	0.051	21.036	***
Ab5 ← ER	0.876	0.052	20.818	***
Ab6 ← ER	0.825			
RB → AB	0.301	0.056	5.290	***
Ab8 ← RB	0.857	0.049	19.761	***
Ab10 ← RB	0.830	0.051	19.124	***
Ab11 ← RB	0.837			
SN → BI	0.243	0.051	4.766	***
Sn1 ← SN	0.770			
Sn2 ← SN	0.888	0.067	18.844	***
Sn3 ← SN	0.879	0.063	18.692	***
NB → SN	0.707	0.047	12.868	***
Nb1 ← NB	0.854	0.047	21.433	***
Nb2 ← NB	0.848	0.047	21.192	***
Nb3 ← NB	0.812	0.048	19.857	***
Nb4 ← NB	0.847			
PBC → BI	0.307	0.058	5.861	***
Pbc1 ← PBC	0.741			
Pbc2 ← PBC	0.884	0.073	17.225	***
Pbc5 ← PBC	0.873	0.072	17.132	***
IPBC→PBC	0.261	0.072	4.329	***
Ipbc1 ← IPBC	0.534	0.088	9.664	***
Ipbc3 ← IPBC	0.882	0.123	11.717	***
Ipbc4 ← IPBC	0.702			
EPBC→PBC	0.299	0.045	5.082	***
Epbc1 ← EPBC	0.781	0.052	17.327	***
Epbc2 ← EPBC	0.812	0.054	18.028	***
Epbc3 ← EPBC	0.862			

The analysis revealed that attitude toward the behavior, subjective norm, and perceived behavioral control all have a direct effect on behavioral intention, with attitude toward the behavior exerting the strongest. Additionally, factors such as evaluation of results, result belief, normative belief, inhabitant factor, and environmental factors were found to have indirect effects on behavioral intention. The strength of these indirect effects was in the following order: normative belief (0.149) > evaluation of results (0.112) > inhabitant factor (0.106) > result belief, and (0.103) > environmental factor (0.079).

#### 3.4.1 Attitude toward the behavioral intention and its influencing factors

Rural residents’ willingness to seek primary care is positively influenced by their attitude toward the behavior. According to Table 7, the path coefficients for the three observed variables—whether primary care organizations are perceived as beneficial for treatment (Ab1), whether they can meet basic healthcare needs (Ab2), and residents’ perceptions of the cost of primary care (Ab3)—are 0.781, 0.824, and 0.869, respectively, which are all significant at the 1% level. This indicates that, in shaping rural residents’ willingness to seek primary care, attitude toward the behavior is objectively affected by the combined effects of Ab1, Ab2, and Ab3, with Ab3 (perceptions of cost) being the most significant factor.

Among the latent variables affecting rural residents’ attitude toward the behavior, the path coefficient for evaluation of results (0.386) is greater than that for result belief (0.301), both significant at the 1% level. This confirms that hypotheses H2 and H3 are valid, with the evaluation of results being the most important factor in shaping rural residents’ attitudes toward primary care.

As shown in Table 7, the factor loading coefficients for all observed variables related to the evaluation of results are greater than 0.6 and significant at the 1% level, which indicates that residents’ satisfaction with the reasonableness of treatment costs (Ab4), level of medical care (Ab5), and physician–patient communication (Ab6) significantly contribute to the evaluation of results, thereby influencing the willingness to seek primary care through attitudes toward the behavior. It has also been proven that only by improving the three aspects of satisfaction, including Ab4, Ab5, and Ab6, can the evaluation of results of rural residents’ primary be improved, subsequently fostering a more positive attitude toward it.

In addition, the analyses show that the path coefficients for “trust in doctors’ medical skills (Ab8), trust in medicines (Ab10), and trust in the medical environment (Ab11) were 0.857, 0.830, and 0.837, respectively, suggesting that rural residents’ result beliefs are influenced by the combined effects of Ab8, Ab10, and Ab11, with trust in doctors’ medical skills (Ab8) being the most critical factor in shaping result belief.

#### 3.4.2 Subjective norm of behavioral intention and its influencing factors

Rural residents’ willingness to seek primary care is positively influenced by subjective norms. According to the results of the model, the path coefficients for the three observed variables—the extent to which access to places of care is entirely at the residents’ discretion (Sn1), others’ approval rating of residents’ choice of primary care for medical care (Sn2), and the extent to which residents are willing to listen to others when choosing primary care (Sn3)—were 0.770, 0.888, and 0.879, respectively. All are significant at the 1% level, which indicates that the subjective norms are jointly affected by Sn1, Sn2, and Sn3, with Sn2 being the most significant factor in forming subjective norms among rural residents.

The path coefficient of normative belief affecting the subjective norm of rural residents is 0.707 and is significant at the 1% level, confirming hypothesis H5. Among the four observed variables for normative belief, the factor loading coefficients for family support for primary care (Nb1), friends’ support for primary care (Nb2), recognition of the graded diagnosis and treatment system (Nb3), and recognition of family doctor service contracts (Nb4) are all higher than 0.7. This indicates that Nb1, Nb2, Nb3, and Nb4 significantly influence normative beliefs, which in turn act on subjective norms, ultimately affecting rural residents’ willingness to seek primary care. Of these, Nb1 (family support) is the most influential factor in shaping normative belief.

#### 3.4.3 Perceived behavioral control of behavioral intention and its influencing factors

Rural residents’ willingness to prioritize primary care for their initial visit is positively influenced by perceived behavioral control. The three observed variables related to perceived behavioral control are all significant at the 1% level, with loadings exceeding 0.7. These variables—convenience of seeking medical treatment in primary care (Pbc1), primary care medical insurance payment rate (Pbc2), and percentage of contracted family doctors (Pbc5)—jointly influence perceived behavioral control, which in turn affects rural residents’ willingness to choose primary care centers for their first visit.

This indicates that when rural residents sign up with a family doctor, the convenience of seeking medical treatment and the higher rate of medical insurance coverage strengthen perceived behavioral control, resulting in a stronger willingness to seek primary care.

Regarding control belief within perceived behavioral control, the path coefficient for environmental factors (0.299) is greater than that for inhabitant factors (0.261), both significant at the 1% level. This confirms that hypotheses H8 and H9 are valid, suggesting that rural residents perceive environmental factors as having a greater impact on perceived behavioral control.

As shown in Table 7, the factor loading coefficients for residents’ age (Ipbc1), average annual disposable income (Ipbc3), and health status (Ipbc4) are 0.534, 0.882, and 0.702, respectively, which significantly affect the inhabitant factor, influencing rural residents’ willingness to seek primary care through perceived behavioral control, with Ipbc3 having the greatest impact.

However, the factor loading coefficients for distance to primary care (Epbc1), accessibility to primary care (Epbc2), and time spent in primary care (Epbc3) were 0.781, 0.812, and 0.862, respectively. These findings indicate that Epbc1, Epbc2, and Epbc3 influence perceived behavioral control through environmental factors, subsequently affecting rural residents’ willingness to seek primary care. Among these, Epbc3 (time spent) has the most significant effect.

## 4 Discussion

This study empirically analyzes the willingness of 415 rural residents in Deqing County, China, to seek primary care and the factors influencing this behavior, using the Theory of Planned Behavior and Amos 22.0 software. The research results show that 86% of the 415 rural residents surveyed are willing to seek primary care. Additionally, 80.24% of the rural residents believe that the proportion of medical insurance coverage for primary healthcare institutions is higher, while 79.76% of the rural residents consider the costs at primary healthcare institutions to be lower. Notably, 92.84% of the rural residents have already experienced primary care services.

The results suggest that the vast majority of rural residents are inclined to prioritize primary care, which is consistent with our expectation of choosing Deqing County as the study sample. However, a subset of residents exhibits a lower motivation to opt for primary care, which may, to some extent, hinder the effective implementation of the graded diagnosis and treatment system.

The empirical analysis confirms that rural residents’ willingness to seek primary care is directly influenced by attitude toward the behavior, subjective norm, and perceived behavioral control and indirectly influenced by evaluation of results, result belief, normative belief, inhabitant factor, and environmental factor. This provides a comprehensive explanation of the formation mechanism of rural residents’ willingness to utilize primary care, aligning with the theoretical expectation of this study. The specific conclusions are as follows:

① Rural residents’ willingness to seek primary care is positively and significantly influenced by attitude toward the behavior, subjective norm, and perceived behavioral control, with attitude toward the behavior exerting the strongest influence. ② Attitude toward the behavior: When rural residents incur lower medical expenses and have greater trust in the medical environment and doctors, their positive behavioral attitudes significantly enhance their willingness to choose primary care as their first option. This finding is consistent with the studies of Zhang et al. (18), Miao et al. (21), and Wang et al. (22). ③ Subjective norm: When rural residents perceive strong support from family members for their choice of primary care, along with a high level of acceptance of the graded diagnosis and treatment system and family doctor contracting services, the subjective norms constructed on this basis significantly promote the formation of willingness to seek primary care. This aligns with the findings of Yang et al. (16), Gao and Yang(17), and Zhao et al. (28). ④ Perceived behavioral control: Factors such as the proportion of health insurance coverage, whether residents are signed up with a family doctor, and the time spent accessing primary healthcare services significantly influence rural residents’ willingness to utilize primary care. These results are consistent with the studies of Li et al. (20), Li et al. (23), and Hu et al. (25).

Based on the study’s findings, the government can improve rural residents’ willingness to prioritize primary care through three key intervention pathways:

① Addressing low motivation related to attitude toward the behavior.

Measures should be taken to strengthen the evaluation of results and result belief.

First, optimize the layout of medical resources, improve their distribution and utilization efficiency, and promote the equalization of primary healthcare service capacity (51).

Second, increase financial investment in primary healthcare institutions, reasonably reduce primary care costs, and alleviate the financial burden on rural residents seeking medical treatment (52).

Third, strengthen the development of primary healthcare teams, enhance the professional skills training of healthcare personnel, and improve their professional standards.

Fourth, improve the mechanism for ensuring medication supply in primary care, increase the availability of medicines, reduce disparities in their usage, and implement a zero-differential-rate policy for medicines.

Fifth, enhance the service orientation of primary healthcare institutions, create a safe and comfortable medical environment, and build patient trust (53).

② Improving the influence of subjective norms: Measures should focus on strengthening normative beliefs.

Use multiple media channels to increase policy awareness, raise rural residents’ understanding of the graded diagnosis and treatment policy, and deepen their knowledge of primary healthcare institutions.

Increase recognition of family doctors and support from family and friends to further guide rural residents toward choosing primary care (54).

③ Enhancing perceived behavioral control: Measures should aim to strengthen both inhabitant and environmental factors. First, raise rural residents’ incomes, improve the level of basic medical insurance, and expand contracted family doctor services to effectively address the issue of high medical costs.

Second, rationally plan the construction of primary healthcare institutions, improve their accessibility, and streamline the consultation process at the primary level to save patients’ time.

By constructing a theoretical model and empirically analyzing data from 30 administrative villages in Deqing County, China, this study provides targeted insights into how to improve primary care rates in rural areas. However, some limitations remain. The study does not consider conditions in other regions, and due to variations in the implementation of the graded diagnosis and treatment system across different areas, there may be significant differences in patients’ willingness and behavior regarding primary care. In addition, time and financial constraints posed challenges to the data collection process, leading to a limited survey sample and potential selection bias. Therefore, future studies could address these issues by conducting comparative analyses with larger sample sizes to obtain more precise results.

## Data Availability

All data generated and analysed during the course of this study are available from the corresponding author upon request. Requests to access these datasets should be directed to Xueqing Zhu: 1310894218@qq.com.
